# SAFEGUARDING OLDER PEOPLE FROM FINANCIAL ABUSE: EXPERIENCES OF IRISH BANKS

**DOI:** 10.1093/geroni/igz038.2132

**Published:** 2019-11-08

**Authors:** Amanda A Phelan, Deirdre O’Donnell, Sandra McCarthy

**Affiliations:** University College Dublin, Dublin, Ireland, Ireland

## Abstract

Financial abuse is a significant issue for older populations and was identified as that most common form of maltreatment in a 2010 Irish prevalence study (Naughton et al. 2010). This study examined how abuse was experienced and responded to by staff in five Irish banks. A mixed method approach was used: online survey (n=898) amd semi structured interviews (n=25) Findings from the survey data demonstrate that more than half of the respondents (66.5%) had previously suspected a customer to be experiencing some form of financial abuse. There was a high index of suspicion to the five scenarios presented to the staff. Findings from the interviews demonstrate the complexity and wide variations of case experiences of bank managers and the National Safeguarding Committee. These include being financially abused in the context of undue influence, scams, fraud and some cases described the naivety or potential naivety of some customers who may have capacity challenges, engage with strangers through social engineering scams, share PINs or open bank accounts without fully understanding the consequences. All staff had some experience of a suspicion of financial abuse in older people and employed various strategies to respond to their suspicions. We focused our recommendations in two areas: Bank level responses-inter-sectorial collaboration, education and training, a vulnerable adult champion, having a choice of banking methods of engagement, direct client communication and enhancements within the Central bank of Ireland. Macro level responses were increasing public awareness, raising the profile of financial abuse as a crime and enhancing safeguarding legislation

